# N-Myc and STAT Interactor regulates autophagy and chemosensitivity in breast cancer cells

**DOI:** 10.1038/srep11995

**Published:** 2015-07-06

**Authors:** Brandon J. Metge, Aparna Mitra, Dongquan Chen, Lalita A. Shevde, Rajeev S. Samant

**Affiliations:** 1 Department of Pathology, University of Alabama at Birmingham, Birmingham, AL, USA; 2 Comprehensive Cancer Centre, University of Alabama at Birmingham, Birmingham, AL, USA; 3 Division of Preventive Medicine, University of Alabama at Birmingham, Birmingham, AL, USA; 4 Mitchell Cancer Institute, University of South Alabama, Mobile, AL, USA

## Abstract

We have previously reported that expression of NMI (N-myc and STAT interactor) is compromised in invasive breast cancers. We also demonstrated that loss of NMI expression promotes epithelial-mesenchymal-transition and results in enhanced invasive ability of breast cancer cells. Additionally we had demonstrated that restoration of NMI expression reduced breast cancer xenograft growth and downregulated Wnt and TGFβ/SMAD signaling. Here we present our observations that NMI expression drives autophagy. Our studies were promoted by our observation that NMI expressing breast cancer cells showed autophagic vacuoles and LC3 processing. Additionally, we found that NMI expression increased the cisplatin sensitivity of the breast cancer cells. Our mechanistic investigations show that NMI prompts activation of GSK3-β. This multifunctional kinase is an upstream effector of the TSC1/TSC2 complex that regulates mTOR signaling. Inhibition of GSK3-β activity in NMI expressing cells activated mTOR signaling and decreased the cells’ autophagic response. Additionally we demonstrate that a key component of autophagy, DNA-damage regulated autophagy modulator 1 (DRAM1), is regulated by NMI. Our TCGA database analysis reveals concurrent expression of NMI and DRAM1 in breast cancer specimens. We present evidence that NMI sensitizes breast cancer cells to cisplatin treatment through DRAM1 dependent autophagy.

Autophagy, (PCD II: programmed cell death II) has been known to play a part in cell survival and apoptosis. Conditions like stress, starvation, and hormone treatment induce the autophagic program^1^,^2^,^3^. Autophagy is a cellular recycling/scavenging process that delivers cytoplasmic components to the lysosomes for degradation. During this process double-membrane vesicles form around portions of the cytoplasm and ultimately fuse with the lysosomes^4^. The connection between autophagy and apoptosis remains poorly understood but it appears increasingly evident that there is a molecular cross-talk between these two pathways^5^,^6^. A basal level of autophagy is present even in non-starved cells to aid in the clearance of misfolded or ubiquitylated proteins^7^.

NMI [N-myc (and STAT) interactor] is an interferon-γ inducible gene product that interacts with several key molecules in cancer cell signaling such as C-MYC, N-MYC, STATs, SOX10 and TIP60^8^,^9^,^10^,^11^,^12^. Previous studies from our group have determined that NMI expression is notably reduced during progression of advanced, invasive breast tumors^13^,^14^,^15^. We have also demonstrated that loss of NMI expression disables negative regulatory control over TGFβ-SMAD signaling and promotes epithelial-mesenchymal-transition (EMT)^13^. Furthermore we noticed that restoring NMI expression in tumorigenic and metastatic cell lines reduced their tumor xenograft growth rates accompanied by suppression of the Wnt/β-catenin signaling pathway^16^. The Wnt/β-catenin signaling and autophagy pathways play important roles during development, tissue homeostasis and tumorigenesis. The Wnt/β-catenin signaling pathway has also been shown to negatively regulate both basal and stress-induced autophagy^17^.

Here we describe our findings that show a novel role of NMI in prompting autophagic induction of breast cancer cells through a GSK3β signaling cascade. Notably, we show that NMI regulates DRAM1, one of the key players in completion of the autophagic program^18^. We demonstrate that loss of NMI reduces the autophagy responsiveness of breast cancer cells and renders them more resistant to chemotherapeutic treatment.

## Materials and Methods

### Cell Culture and Stable Cell Line Generation

MCF10CAcl.d is an isogenic, metastatic cell line derived from *in vivo* passages of MCF10AT (tumorigenic) in nude mice^19^,^20^. This cell line was obtained from the Barbara Ann Karmanos Cancer Institute (Detroit, MI, USA). MCF10CAcl.d cells were grown in DMEM/F-12 (Life Technologies) media supplemented with 5% Horse Serum (Life Technologies), 10 μg/ml cholera toxin(Sigma), 10 μg/ml insulin, 25 ng/ml EGF(Sigma), and 500 ng/ml hydrocortisone (Sigma). T47D cells were grown in RPMI-1640 media (Life Technologies) supplemented with 10% FBS (Atlanta Biolgicals), 1% sodium pyruvate (Life Technologies), and 10 μg/ml insulin. MDA-MB-231 cells were grown in DMEM/F-12 media supplemented with 5% FBS and 1% sodium pyruvate.

Stable expressors of NMI in MCF10CAcl.d and MDA-MB-231 are described previously^13^,^16^. T47D breast cancer cells silenced for NMI are described previously^13^.

### Plasmid Constructs and Transfection

EGFP-LC3 construct was obtained from Addgene (plasmid #24920). HA-GSK3-β-K85A, a kinase dead GSK3-β mutant, was obtained from Addgene (plasmid #14755). Lipofectamine 2000 was used for transfection of HA-GSK3-β-K85A as per the manufacture’s protocol. pEGFP-LC3 was transfected into cells using Lipofectamine 2000 and visualized with fluorescence microscopy 48 hours post- transfection. siRNA specific to DRAM1(siGENOME 55332), along with a corresponding non-targeting negative control was obtained from Thermo Fisher. siRNA pools were transfected at 100 nM using Lipofectamine 2000 and assayed for knockdown at 48 hours post-transfection; or subjected to drug treatment 24 hr post transfection.

### Western Blotting

Cells washed in ice-cold phosphate-buffered saline (PBS) were incubated in ice-cold lysis buffer for 30 min (1% NP-40, 150 mM NaCl, 50 mM Tris, with protease and phosphatase inhibitors). Cell lysates were kept on ice for 1 hr, and then cleared of any debris by spinning at 13,000 RPM for 10 min at 4 °C. Protein concentration was determined using Precision Red reagent by measuring the O.D. at 600 nm and calculated based on 1 O.D. 600 nm is equivalent to 125 μg protein per ml (Cytoskeleton, Denver, CO). 30 μg total protein was resolved using SDS-PAGE and transferred to PVDF membrane. Membranes were blocked in 5% non-fat dry milk in Tris-buffered saline with 0.1% Tween-20 and primary antibodies were added overnight at 4 °C. Membranes were washed in TBST and incubated with specific HRP-conjugated secondary antibodies. The following antibodies were used: Anti-Nmi (1:5000, Sigma Catalog# WH0009111M1), Anti-LC3B (1:1000, Cell Signaling Catalog# 3868), Anti-p62 SQSTM (1:1000, Cell Signaling Catalog# 8025), Anti-p70S6K (1:1000, Cell Signaling Catalog# 9202), Anti-p70S6K phospho Thr389 (1:1000, Cell Signaling Catalog# 9205), Anti-GSK3-β (1:1000, Cell Signaling Catalog# 9315), Anti-GSK3β-phospho-ser9 (1:1000 , Cell Signaling Catalog# 9323), Alpha/Beta Tubulin (1:5000, Cell Signaling Catalog# 2148), Anti-GAPDH (1:10,000, Cell Signaling Catalog# 2118), Anti-DRAM1 (1:500, Sigma Ab-2 Catalog# PRS4035), Anti-Beclin-1 (1:1000, Cell Signaling Catalog# 3738). Western blots were developed using SuperSignal West Dura and exposed to CL-X-posure film (Thermo Scientific).

Densitometry analysis was performed using AlphaEaseFC software (Genetic Technologies, Miami, FL). Analysis was done using GAPDH, Alpha/Beta Tubulin, or where appropriate total p70S6K or GSK3-β for determining fold changes in protein expression.

### Starvation of Cells

To induce autophagy, cells were incubated under nutrient starved conditions. Briefly, cells were plated at 2×10^5^ cells per well in a 6 well plate. The day after plating, cells were washed 3 times in PBS (37 °C); Earl’s Balanced Salt Solution (EBSS; Life Technologies) was added to cells. Where indicated, 20 μM chloroquine was also added with EBSS treatment to block the cleavage of LC3. Cells were incubated for 1 h at 37 °C in humidified CO_2_ incubator after which they were harvested. Where indicated, cells were treated with 100 nM Bafilomycin A1 (Sigma) for 4 hours prior to treatment with EBSS for 1 hour.

### Drug Treatment and MTS Assay

Cells were plated in 96 well tissue culture treated plates at a density of 5×10^3^ cells per well. Fresh complete media containing either 0.1 μM or 0.5 μM Doxorubicin (Calbiochem), or 10 μM or 25 μM Cisplatin (Sigma) was added to cells. The cells were incubated for either 48 h or 72 h and cell viability was assessed using MTS assay (Promega).

In some experiments cells were initially transfected with siRNA prior to a drug treatment. Briefly, cells were seeded in a 96 well plate at a density of 1×10^4^ cells per well and transfected as previously described with siRNA specific to DRAM1. 24 hours post transfection the media was changed to media containing 10 μM or 25 μM cisplatin and the cells were incubated for an additional 48 hours. Cell viability was assessed by MTS assay.

### Labeling cells with LysoTracker

Acidic compartments were labeled with 1 μM LysoTracker (Lysotracker Green DND-26, Molecular Probes) in the culture media for 15 minutes at room temperature. After incubation, cells were washed with PBS and immediately analyzed by fluorescence microscopy.

### Fluorescent Autophagy Detection

Autophagy activity was determined using the Cyto-ID Autophagy Detection kit (Enzo Life Sciences, Farmingdale, NY) as per manufacturer’s protocol. Briefly, cells were seeded in a 6 well plate, and the following day cells were starved using EBSS as previously described. After starvation cells were labeled with Cyto-ID and Hoechst 33342 dyes for 30 minutes at 37 °C and then washed in PBS. Images were capture with Eclipse Ti-U using the same exposure times for all images acquired (Nikon Instruments Inc. Melville, NY).

### Immunohistochemistry Staining

Paraffin embedded blocks of tumors resected from MDA-MB-231 vector and NMI xenograft models were stained for both LC3B and NMI. Briefly, blocks were deparaffinzed and re-hydrated followed by citrate antigen retrieval. Primary antibodies were diluted in 1% BSA in PBS at the following dilutions: NMI 1:10,000 (Sigma), and LC3B 1:4000 (Cell Signaling) and incubated overnight at 4 °C. Slides were then washed and the Dako En Vision + HRP kit (Dako) was subsequently used. Slides were counterstained with Harris Hematoxylin and then mounted with Cytoseal. Images were captured with Eclipse Ti-U. (Nikon Instruments Inc. Melville, NY.)

### TCGA Data analysis

The Cancer Genome Atlas (TCGA) data portal of National Cancer Institute (NCI) was used to download data of the breast invasive carcinoma samples (n = 1,100). The RNAseqV2 level 3 data that includes FPKM (fragments per kilobase of exon per million fragments mapped)-normalized gene level data were used before statistical analysis by using Partek Genome Suite (PGS, St. Louis, MI). In addition, idf file and sdrf files were also downloaded for sample mapping and annotation. All samples were divided into three groups (high or H, n = 366; medium or M, n = 368; and low or L, n = 366) based on NMI expression levels. The same strategy was applied to divide these 1,100 samples into DRAM1-high(H), -medium(M), and -low(L) groups by using DRAM1 expression levels. Those samples categorized as both NMI-H and DRAM1-H, or NMI-L and DRAM1-L (n = 273) were selected and correlated with triple negative (TNBC) phenotype. Hierarchical clustering using Euclidean dissimilarity matrix was performed after z-normalization. Correlations of gene expression were performed by using Pearson correlation method.

### Statistical Analysis

Data are expressed as the mean ± S.E.M. from at least three independent measurements. Unless otherwise indicated the differences between groups was analyzed by the Student’s *t*-test.

## Results

### NMI potentiates autophagy

**Figure 1:**
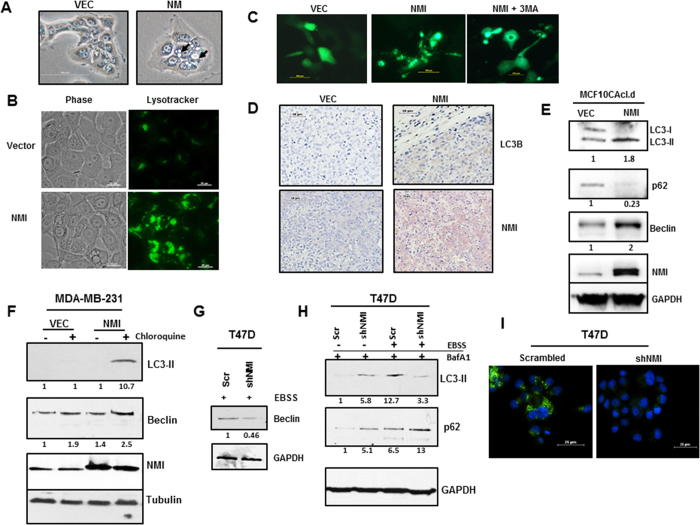
NMI alters autophagy in breast cancer. (**A**) MCF10CAcl.d vector or NMI cells visualized with phase contrast, arrows indicate vacuolated structures in NMI cells. (**B**) MCF10CAcl.d Vector and NMI cells were stained with 1 μM Lysotracker to visualize acidic vacuoles. (**C**) MCF10CAcl.d Vector and NMI cells transfected with LC3-GFP or in combination with 3 MA, images taken at 48 hours after transfection (scale bar = 100 μm). (**D**) Tumor xenografts of MDA-MB-231 Vector or NMI stained for LC3B or NMI. Images taken at 20X (scale bar = 50 μm) (**E**) Immunoblot analysis of markers for autophagy of LC3B, p62 and Beclin in MCF10CAcl.d vector and NMI cells. Full-length blots are presented in Supplementary Figure 1. (**F**) Stress induced autophagy of MDA-MB-231 vector and NMI cells treated with EBSS for 1 hour with or without the addition of 20 μM chloroquine. Full-length blots are presented in Supplementary Figure 2. (**G**) T47D cells silenced for NMI treated with EBSS and Beclin levels were determined using immunoblot analysis. (**H**) T47D cells silenced for NMI treated in 100 nM BafA1 then treated with EBSS for 1 hour to assay effects on autophagy using immunoblot analysis of LC3B and p62. Full-length blots are presented in Supplementary Figure 3. Fold change determined by densitometry analysis is indicated below each lane of corresponding blots. (**I**) T47D silenced cells were EBSS starved and stained using Cyto-ID autophagy detection kit. (scale bar = 25 μm)

Relative to control cells, NMI expressing MCF10CAcl.d cells appear morphologically distinct, displaying increased number of vacuoles when visualized under phase contrast (Fig. 1A). These vacuoles positively stained with Lysotracker, indicating the presence of acidic lysosome structures (Fig. 1B). During autophagy, LC3 gets lipidated and processed from LC3-I to LC3-II. The amount of LC3-II can be correlated to number of autophagic vesicles in the cell. We used exogenous LC3-GFP expression to confirm changes in autophagy in NMI expressing cells. A distinct punctate GFP localization indicative of processed LC3 was observed in MCF10CAcl.d-NMI cells as compared to diffuse GFP localization in the vector cells. Upon treatment of the NMI expressing cells with a known autophagy inhibitor, 3-methyladenine (3MA), the punctate GFP localization became a more diffuse cytoplasmic stain similar to that of the vector control cells (Fig. 1C).

In our previous work we have demonstrated that NMI alters breast cancer progression in xenograft tumor models, specifically that MDA-MB-231 cells restored for NMI expression have markedly delayed tumor growth and metastases^16^. We made use of paraffin embedded tumors resected from vector control and NMI expressing xenografts and stained the tissues for LC3. A clear distinction can be noted in that the vector tumors were devoid of any LC3 stain, whereas there was prominent indication of staining in the NMI expressing tumors (Fig. 1D).

We further investigated if NMI was potentiating autophagy by investigating the changes in expression of key autophagy markers. In MCF10CAcl.d cells expressing NMI, LC3-I is almost completely processed to LC3-II and there is a concomitant gain of Beclin expression (Fig. 1E). p62 is a receptor for cargo such as ubiquitinated proteins that are destined to be degraded by autophagy. The p62 protein is able to bind ubiquitin and also to LC3, thereby targeting the autophagosome and facilitating clearance of ubiquitinated proteins^21^. We assayed for changes in p62 in NMI expressing cells and found that p62 levels are markedly decreased as compared to vector controls further confirming autophagy modulation via NMI (Fig. 1E).

Interestingly, in some of the cell lines restored for NMI expression we did not see the same basal level changes in autophagy. This may possibly be due to additional alterations in the signaling pathways that result in driving autophagy differently across different cell lines. To further ascertain the role of NMI in autophagy, MDA-MB-231 NMI expressing cells were subjected to EBSS treatment to induce autophagy in addition to chloroquine to allow for increased accumulation of LC3. We noticed that in vector control cells there was no appreciable increase in LC3-II after EBSS treatment; however, in NMI cells there was a dramatic increase in the level of LC3-II present in these cells after treatment. Additionally, NMI expressing cells showed increased levels of Beclin upon starvation as compared to vector control (Fig. 1F). These results suggest that NMI may sensitize cells to autophagy in response to certain stress conditions.

As a converse approach, we made use of a T47D breast cell line that had been stably silenced for NMI to determine the effect of NMI silencing on autophagy. We did not notice any significant difference at the basal autophagic level. However, when growth media was replaced with EBSS, the cells showed blunted induction of autophagy as evidenced by a lack of Beclin increase in NMI silenced cells (Fig. 1G). Furthermore, autophagic flux was altered in silenced cells as demonstrated by dramatic reduction in the processing of LC3-II with a concomitant increase in the level of p62 compared to the control cells when treated with Bafilomycin A1 during starvation (Fig. 1H). In addition, we made use of a fluorescence system for tracking autophagy such that when autophagy is activated cells will emit bright green fluorescence. When T47D scrambled cells were treated with EBSS a bright punctate fluorescence is detected; however, in NMI silenced there is minimal fluorescence detected (Fig. 1I). Collectively, these results demonstrate that NMI levels affect the autophagy process in breast cancer cells.

### NMI alters mTOR signaling via GSK3β

**Figure 2:**
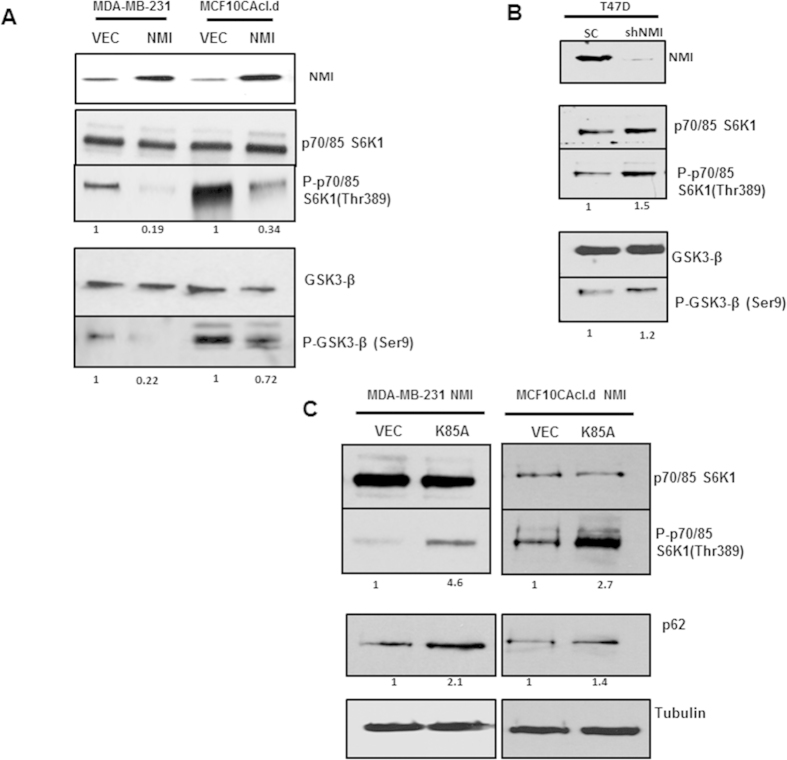
mTOR/GSK3β signaling axis is dysregulated by NMI. (**A**) Immunoblot analysis of MDA-MB-231 and MCF10CAcl.d Vector and NMI for phosphorylated p70S6K1 and total p70S6K1 as well as GSK3β-ser9 phosphorylation. Full-length blots are presented in Supplementary Figure 4,5. (**B**) T47D NMI silenced cells show a reversion in p70SK1 and GSK3β phosphorylation as determined by western blot. Full-length blots are presented in Supplementary Figure 6. (**C**) MDA-MB-231 and MCF10CAcl.d NMI expressing cells transfected with a dominant negative GSK3β kinase dead mutant (K85A). Immunoblot analysis was done 48 hours post transfection for p70S6K1 phosphorylation and p62. Full-length blots are presented in Supplementary Figure 7,8. Fold change determined by densitometry analysis is indicated below each lane of corresponding blots.

The mTOR signaling pathway shows prime involvement in negatively influencing autophagy through regulation of the ULK-1/ ATG13 / FIP200 protein complex^22^. p70S6K1 is a direct target of the mTORC1 complex that plays a role in cap dependent translation, mRNA biogenesis, and ribosome protein translation^23^. To determine if mTOR signaling was negatively influenced in response to NMI expression, we assayed for phosphorylation changes in p70S6 kinase. In comparison to vector controls, NMI expressing MCF10CAcl.d and MDA-MB-231 showed marked decrease in phosphorylated p70S6K1, indicating that NMI expression had a negative impact on mTOR signaling. This observation is consistent with the increased autophagy response (Fig. 2A). Conversely, NMI silencing in T47D cells resulted in increased levels of phosphorylated p70S6K1 indicating overall activation of mTOR signaling leading to suppression of autophagic response (Fig. 2B). AKT signaling is one of the key drivers of mTOR activation. However, we failed to see activation of AKT (AKT1Ser^478^ phosphorylation) in NMI expressors (data not shown). This raised a possibility that effect of NMI on mTOR signaling may be independent of AKT.

To uncover the details of activity of NMI in abrogating mTOR signaling, we assayed for changes in GSK3β activation. GSK3β is upstream regulator of the TSC1/TSC2 complex which regulates mTOR signaling through mTORC1 based on Rheb GDP-loading^23^,^24^. We found that restoring expression of NMI led to decreased levels of phosphorylated Ser-9-GSK3β; indicating more active GSK3β in these cells (Fig. 2A). Conversely, NMI silencing resulted in increased abundance of phosphorylated Ser-9-GSK3β indicating overall inactivation of GSK3β (Fig. 2B). To further confirm the involvement of GSK3β, we decided to block its activity using a dominant negative, kinase dead GSK3β (GSK3β-K85A). MDA-MB-231 and MCF10CAcl.d cells re-expressing NMI (which have active GSK3β) were transfected with the GSK3β-K85A mutant and probed for changes in p70S6K phosphorylation. Indeed we saw that blocking GSK3β activity was concomitant with an increase in p70S6K phosphorylation indicating the resultant upregulation of mTOR signaling. Additionally, we noticed a simultaneous increase in the level of p62 as a consequence of blocking GSK3β activity in NMI expressing cells (Fig. 2C). These results demonstrate that NMI upregulates autophagy through GSK3β.

### NMI sensitizes cells to Drug Treatment via Autophagy

**Figure 3:**
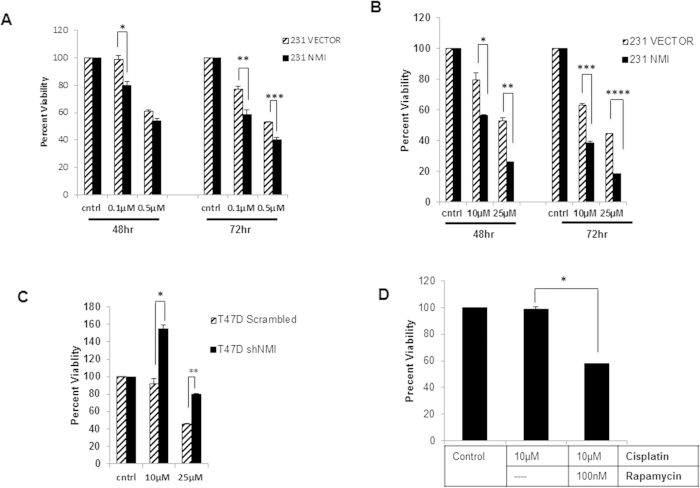
NMI expression increases chemosensitivity (**A**) MDA-MB-231 vector or NMI cells were plated in a 96 well plate in triplicate and treated with either 0.1 μM or 0.5 μM doxorubicin for 48 and 72 hours and MTS was done to measure cell viability. Percent viability is measured as fold change of corresponding vehicle control (*p = .04, **p = .04, ***p = .02) (**B**) Cisplatin treatment of either 10 μM or 25 μM at 48 and 72 hours (*p = .03, **p = .003, ***p = .002, ****p = .0001) (**C**) T47D scrambled or shNMI cells were treated with 10 μM or 25 μM cisplatin for 48 hours and MTS was done to measure cell viability. (*p = .01, **p = .005). (**D**) T47D NMI silenced cells were treated with 10 μM cisplatin alone or in combination with 100 nM rapamycin for 48 hours and MTS was done to measure cell viability (*p = .001).

It has become increasingly clear that autophagy plays a pivotal and context dependent role in impacting drug resistance phenotype of cancer^25^,^26^,^27^. To determine the implications of NMI modulated autophagy in breast cancer, we focused our attention towards understanding the influence of NMI on drug sensitivity. MDA-MB-231 vector or NMI expressing cells were treated with doxorubicin and assayed at various time points for viability. It is evident that cells expressing NMI are approximately 20–30% more sensitive to treatment of doxorubicin compared to vector cells. This response was noted with treatment for 48 hrs and it became more noticeable at the 72 hour time point (Fig. 3A). Platinum based drugs such as cisplatin are being increasingly advocated for metastatic triple negative breast cancer patients^28^,^29^,^30^. Treatment of the MDA-MB-231 vector or NMI expressing cells with cisplatin showed that NMI expression greatly enhances the sensitivity of breast cancer cells to cisplatin. Specifically, 25 μM cisplatin treated NMI cells display a distinctly noticeable 50–60% increase in sensitivity at both 48 and 72 hours (Fig. 3B). To test if absence of NMI would negatively impact the sensitivity, we sought to test the effect of cisplatin on T47D cells silenced for NMI. The scrambled control cells had a very robust sensitivity to cisplatin when treated with 25 μM; however, silenced cells showed a marked resistance to treatment with only a 20% reduction in viability (Fig. 3C). Taken together these results indicate that NMI has a vital role in modulating drug sensitivity.

Previous studies have shown that rapamycin induced autophagy enhances the effect of chemotherapy^31^; therefore we hypothesized that NMI induction of autophagy may be contributing to the increased drug sensitivity. To confirm a role for NMI mediated drug resistance through autophagy, we utilized the T47D cell line silenced for NMI which was resistant to treatment with cisplatin. When these cells were co-treated with cisplatin and rapamycin they showed a remarkable 50% reduction in cell viability (Fig. 3D). These results suggest that NMI has a vital role in modulating the drug response in cells through activation of autophagy.

### NMI modulates DRAM1 and effects Drug Resistance

**Figure 4:**
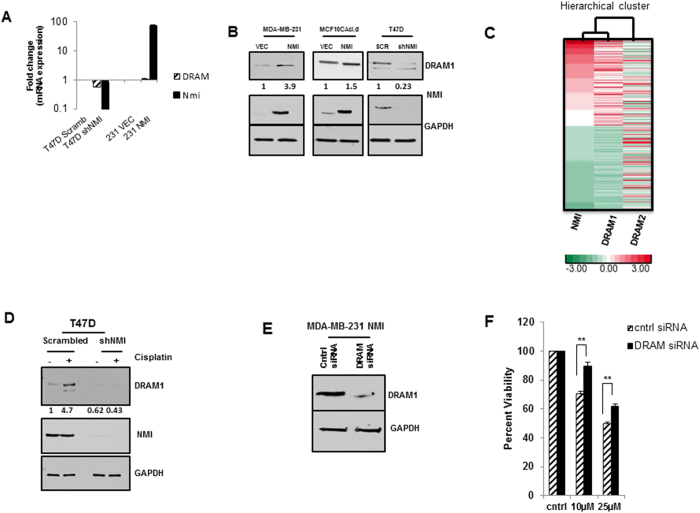
NMI affects chemoresistance *via* DRAM1. (**A**) Quantitative RT-PCR analysis of T47D NMI silenced and MDA-MB-231 NMI cells for DRAM1 and NMI mRNA levels. Data are normalized to GAPDH expression and fold changes in expression (log10) are relative to corresponding vector or scrambled control. (**B**) Immunoblot analysis of DRAM1 expression in NMI cells restored for NMI or silenced for NMI, GAPDH used as loading control. Full-length blots are presented in Supplementary Figure 9, 10, 11. (**C**) TCGA data was analyzed for trends of NMI and DRAM1 expressions (n = 273, NMI-H and DRAM1-H, NMI-L and DRAM1-L) were clustered based on expressions of NMI, DRAM1 and DRAM2 as a control (H = high L = low). Red = high; green = low. (**D**) T47D scrambled and silenced cells were treated with 20 μM cisplatin or DMSO for 24 hours and subjected to immunoblot analysis for DRAM1. Full-length blots are presented in Supplementary Figure 12. Fold change determined by densitometry analysis is indicated below each lane of corresponding blots. (**E**) MDA-MB-231 NMI expressing cells transfected with 100 nM siRNA to DRAM1 and confirmed for knockdown 48 hours post-transfection. Full-length blots are presented in Supplementary Figure 13. (**F**) MDA-MB-231 NMI cells transfected with 100 nM DRAM siRNA or non-target control and were subsequently treated with 10 μM or 25 μM cisplatin for 48 hours. Cell viability was determined by MTS and percent viability is measured as fold change of corresponding vehicle control (*p = .0025, **p = .0015).

Damage–regulated autophagy modulator, DRAM1 was originally identified as an inducer of autophagy that mediates cell death^32^. DRAM1 has also been shown to be involved in mediating cisplatin response^33^. We followed this serendipitous literature lead and explored the possibility that NMI could be affecting cisplatin sensitivity via a DRAM1 dependent autophagy mechanism. We first queried for changes in DRAM1 transcript levels upon expression of NMI or silencing of NMI, and found a minimal positive change in MDA-MB-231-NMI cells; however, DRAM1 transcript was reduced about 2-fold in T47D NMI silenced cells (Fig. 4A). Though at mRNA level the effect of NMI on DRAM1 was cell line dependent, we found a more consistent response at protein level. DRAM1 protein expression dramatically increased in MDA-MB-231-NMI expressing cells as compared to vector controls. MCF10CAcl.d-NMI expressors also showed consistent trend of increased DRAM1 expression. Conversely, a decrease in protein expression of DRAM1 was noted in T47D-NMI silenced cells (Fig. 4B).

To test the relevance of the NMI-DRAM1 co-expression we queried the NCI-Cancer Genome Atlas (TCGA) data base for breast invasive carcinoma samples (n = 1,100). All samples were divided into three groups (high or H, n = 366; medium or M, n = 368; and low or L, n = 366) based on NMI expression level. The same strategy was applied to divide these into DRAM1 high, medium, and low groups by using DRAM1 expression levels. Those samples categorized as both NMI-H and DRAM1-H, or NMI-L and DRAM1-L (n = 273) were analyzed. As seen in Fig. 4C, NMI and DRAM1 show a highly significant correlative expression with correlation coefficient (CC) of 0.71 and p < 3.7e-042. It is important to note that NMI expression does not show a correlation trend (CC of 0.08 and p < 0.162) with a distinct gene DRAM2 (Damage regulated autophagy modulator 2) that has functional overlap with DRAM1 (Fig. 4C).

It has been reported that DRAM1 gene expression is upregulated in response to cisplatin treatment^33^,^34^. Given that NMI appears to modulate the expression of DRAM1, we decided to test the effect of cisplatin treatment on DRAM1 expression in cells silenced for NMI. T47D control cells, treated with 20 μM cisplatin for 24 hours, show a robust upregulation of DRAM1; conversely, in NMI silenced cells DRAM1 expression is not modified by treatment with cisplatin (Fig. 4D). Based on this observation we tested if NMI modulation of DRAM1 may indeed affect breast cancer cells sensitivity to drug treatment. We hypothesized that increased DRAM1 expression in NMI expressing cells was contributing to the increased cisplatin sensitivity seen in these cells; therefore, silencing of DRAM1 would lead to drug resistance in NMI expressing cells. MDA-MB-231-NMI cells, which showed increased levels of DRAM1, were transfected with siRNA to efficiently knockdown DRAM1 (Fig. 4E). Indeed, when DRAM1 was silenced in MDA-MB-231-NMI cells there was a 20% increase in cell viability in response to cisplatin treatment (Fig. 4F) indicating that the cytotoxic effects of cisplatin were diminished. Thus our observations imply that NMI and DRAM1 expression trends may have significance in determining the sensitivity of patients to cisplatin treatment.

## Discussion

Autophagy is a complex cellular process that can serve multiple roles in cell physiology. Its roles are quite dubious particularly in the context of cancer where multiple studies have shown autophagy to serve as a mechanism of cell survival under adverse conditions or a mechanism that prompts cell death^35^,^36^,^37^. In order for tumor cells to establish a niche they must be able to evade many obstacles in a harsh microenvironment where they will be subjected to nutrient stress and hypoxia; in these instances the tumor cells are able to use autophagy to prolong survival^5^,^37^,^38^,^39^. However, the role of autophagy in cancer is quite complex and very context dependent on whether it is pro-survival or facilitates cell death either promoting apoptosis or actively killing cells^35^,^36^. Currently there is no clear consensus on the role of autophagy as it relates to different treatment modalities, there are numerous conflicting reports in the literature regarding whether inhibition of autophagy may facilitate drug therapies i.e. chloroquine or activation of autophagy with agents such as rapamycin is a more effective treatment regimen^40^,^41^,^42^. Our current focus identified a new key modulator of autophagy, NMI, as it was found to enhance autophagy in breast cancer as well as change the drug sensitivity profile of these cells.

Recent studies from our group have shed light on the importance of N-myc interactor, and the impact of its loss on promoting breast cancer progression^13^,^14^,^16^. NMI was initially described as an interferon gamma inducible protein, and interestingly previously reports have also shown that autophagy can be induced by IFNγ in macrophages^43^. We observe increased autophagy in breast cancer cells with NMI expression restored as determined by increased LC3-II processing along with decreased p62 and increased LC3 staining of tumor xenografts of MDA-MB-231-NMI. It is noteworthy that these cells show reduced tumor growth rate^16^. mTOR is the key node of the autophagy regulatory network and acts downstream on the ULK1/ATG13/FIP200 complex which is involved in the direct regulation of autophagosome biogenesis^22^. Our previous work has uncovered a significant negative impact of NMI expression of WNT/β-catenin signaling in breast cancer^14^,^16^. GSK3β is a multifunctional kinase that negatively regulates WNT/β-catenin signaling, but has roles in multiple other signaling cascades, one of which involves its ability to regulate the TSC1/TSC2 complex upstream of mTOR resulting in inhibition of this signaling cascade^23^,^24^. We find that NMI maintains GSK3β in an active state which inactivates mTOR signaling and consequently upregulates autophagy.

Autophagy is a process that not only regulates cell survival but also plays an important role as a clearance mechanism for proteins and large protein complexes^4^,^7^. One of the more interesting aspects of NMI’s function is its role in EMT (epithelial-mesenchymal transition) and ability to modulate EMT through TGFβ signaling^13^. Recent studies of autophagy have shown that it may play a pivotal role in EMT by controlled degradation of key regulatory proteins vital to the EMT program. Work by Lv *et al.* found that two master regulators of EMT, Snail and Twist, could be selectivity degraded via the autophagy machinery and not the proteosome when activated by a novel tumor suppressor DEDD^44^,^45^. These findings elicit some interesting speculation about NMI, regulation of EMT, and autophagy. Regulation of the EMT program is a tightly controlled process in which loss of NMI lifts an inhibitory control on TGFβ signaling^13^. Additionally, it seems likely that induction of autophagy *via* NMI may be another vital element in this process given that master regulators of EMT are susceptible to degradation through the autophagy pathway.

While the role of autophagy and its regulation in cancer cells continues to emerge, studies aim to define optimal strategies to modulate autophagy for therapeutic advantages. Numerous conflicting reports still strive to resolve whether activation or inhibition of autophagy in combination with chemotherapeutic agents will yield the optimal treatment response^5^,^37^. Here we demonstrate for the first time that NMI is able to affect the drug response of breast cancer cells to both doxorubicin and cisplatin, wherein restoring NMI expression yields cells more sensitive to these treatments with about a 50% increase in cell death. Furthermore, in breast cancer cells that have lost expression of NMI we see a significant resistance to treatment. Published reports have shown that in some cases autophagy may also act to sensitize cells to cancer killing agents. In renal cell carcinoma small molecule inhibitors were shown to induce death through an autophagy dependent mechanism^46^. Additionally, a study from Seca *et al.* determined that autophagy induction through miR-21 in leukemia increased chemosensitivity^47^.

We found that NMI levels directly correlated with those of DRAM1 a mediator of autophagy and cell death^32^. DRAM1 encodes a lysosomal membrane protein that is required for the induction of autophagy and has also been found to be downregulated across epithelial cancers^32^. Previous work has shown the importance of DRAM1 in mediating the response to cisplatin, specifically; DRAM1 is induced upon treatment of breast cancer cells with cisplatin^33^. In hepatoma the importance of DRAM1 induced autophagy as a viable therapeutic approach has also been demonstrated^48^. Our current work also highlights the importance of DRAM1 mediating cisplatin drug response, but more importantly, the critical role that NMI plays in this process, as cells silenced for NMI are not able to upregulate DRAM1 in response to cisplatin treatment. Additionally, in cells expressing NMI, silencing of DRAM1 dramatically renders the cells resistant to cisplatin. This indicates the importance of NMI and DRAM1 in determining sensitivity of breast cancer to cisplatin. An interesting clinical perspective has emerged from our analysis of the TCGA database. We clearly notice a significant correlation in co-expression of NMI and DRAM1, which supports our cellular observations.

Over the years, multiple studies have looked at platinum based agents in patients with newly diagnosed metastatic breast cancer or refractory metastatic breast cancer with a definite focus on TNBC^29^,^30^. A recent study presented at ASCO 2014 investigated the benefits of cisplatin administration relative to paclitaxel in TNBC (NCT01982448) since low dose cisplatin has fewer side effects, including lesser renal toxicity^28^,^29^,^30^. From our cohort of 273 breast cancer patients only 39 represented patients with TNBC. This is a small dataset to draw significant conclusions. The recent trends in genomic analysis of patient specimens has made it clear that multiple subgroups of patients exist and if this aspect is not carefully considered, clinical trials yield a mix of responders and non-responders. Based on our functional and mechanistic findings NMI and DRAM1 play key roles in determining the response of breast cancer to cisplatin and we suggest that NMI and DRAM1 levels may offer additional parameters towards these patient considerations. Overall, our studies highlight the importance of autophagy in breast cancer therapy and the need for continued studies in this complex and diverse aspect of cancer biology.

## Additional Information

**How to cite this article**: Metge, B. J. *et al.* N-Myc and STAT Interactor regulates autophagy and chemosensitivity in breast cancer cells. *Sci. Rep.* **5**, 11995; doi: 10.1038/srep11995 (2015).

## Supplementary Material

Supplementary Information
