# Supplementary material for: N-Myc and STAT Interactor regulates autophagy and chemosensitivity in breast cancer cells
Source: Sci Rep. 2015 Jul 6;5:11995. doi: 10.1038/srep11995 (PMC4648342; doi:10.1038/srep11995)
Supplement: Supplementary Information [file srep11995-s1.pdf]

N-Myc and STAT Interactor regulates autophagy and chemosensitivity in breast cancer cells.

Brandon J. Metge[1], Aparna Mitra[4], Dongquan Chen[1,2,3], Lalita A. Shevde[1,2], Rajeev S. Samant[1,2†]

Running title: NMI regulates autophagy

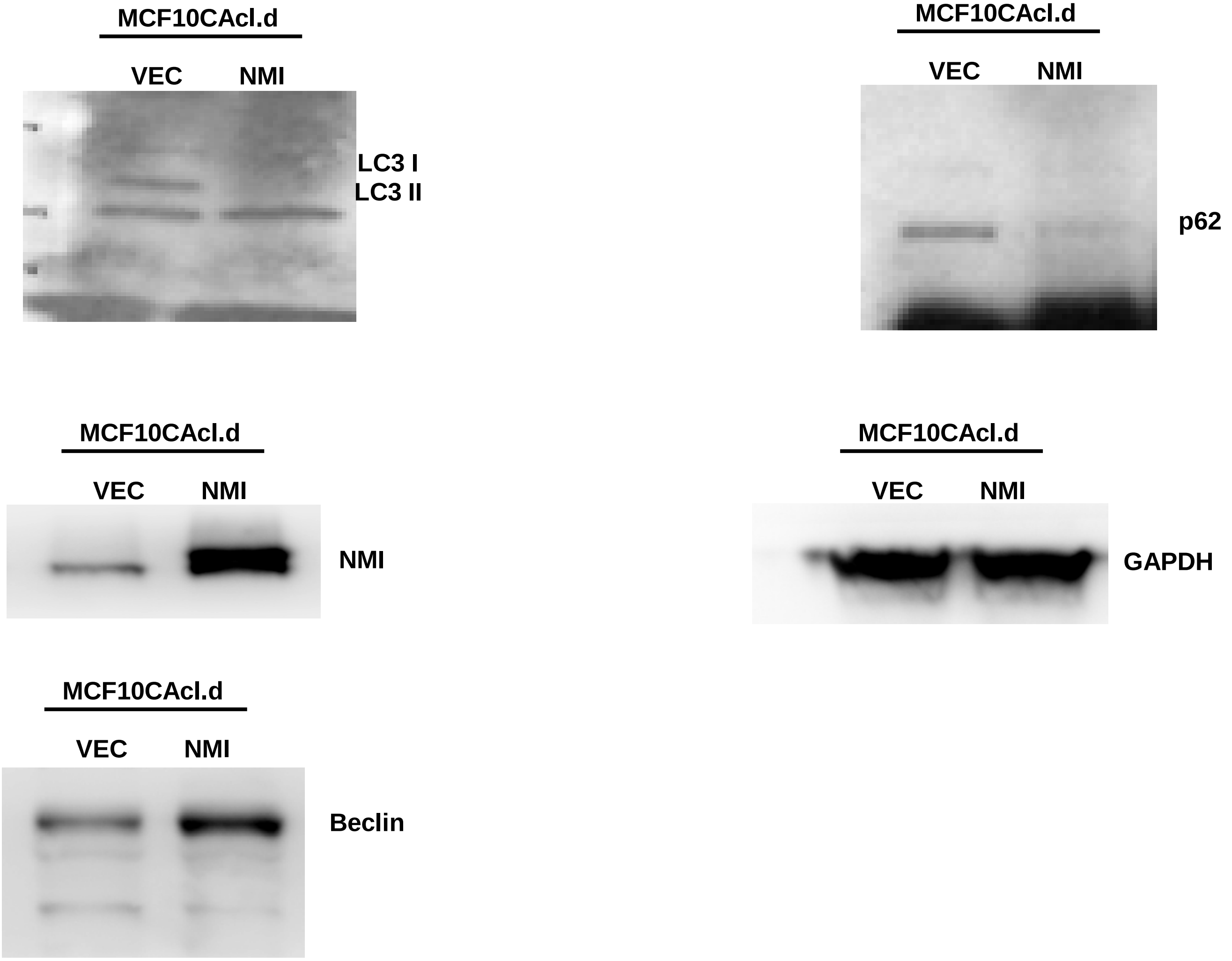

Supplementary Figure 1

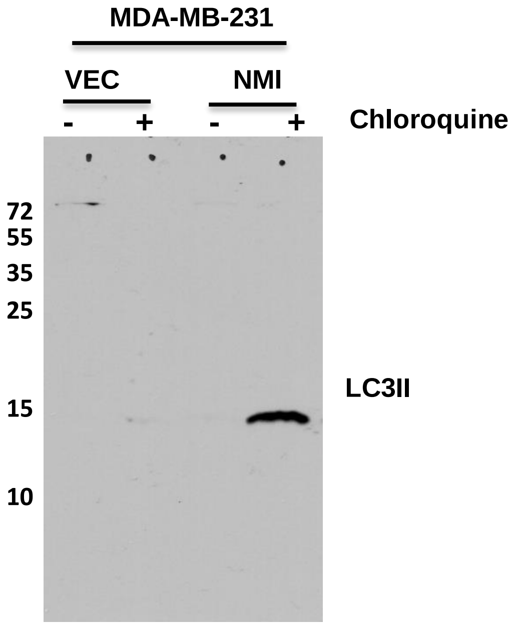

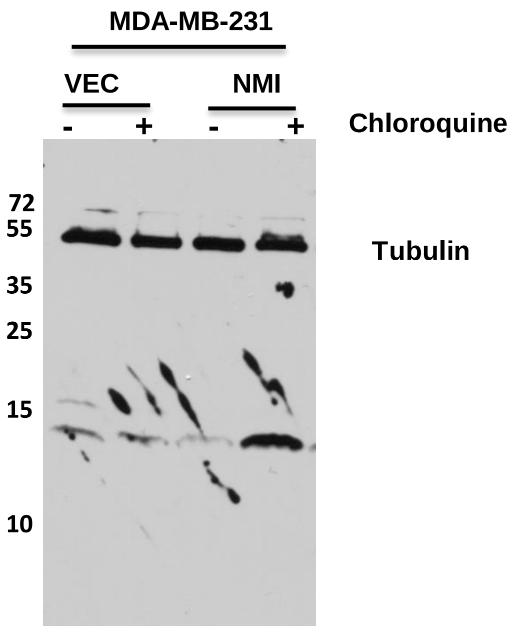

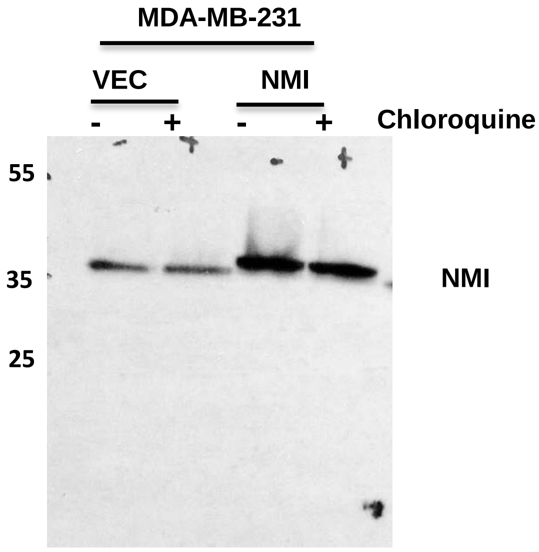

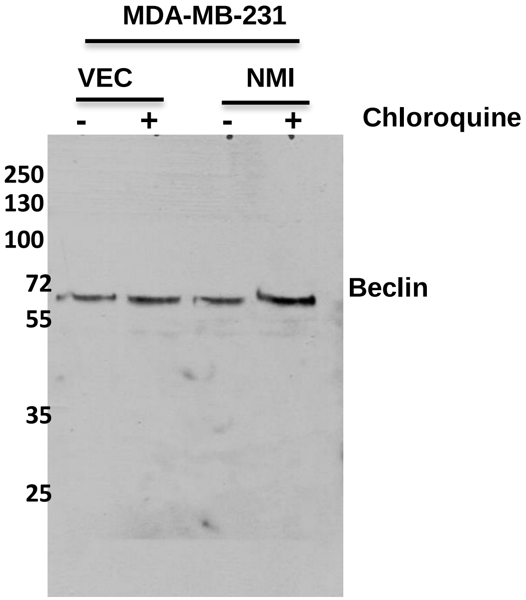

Supplementary Figure 2

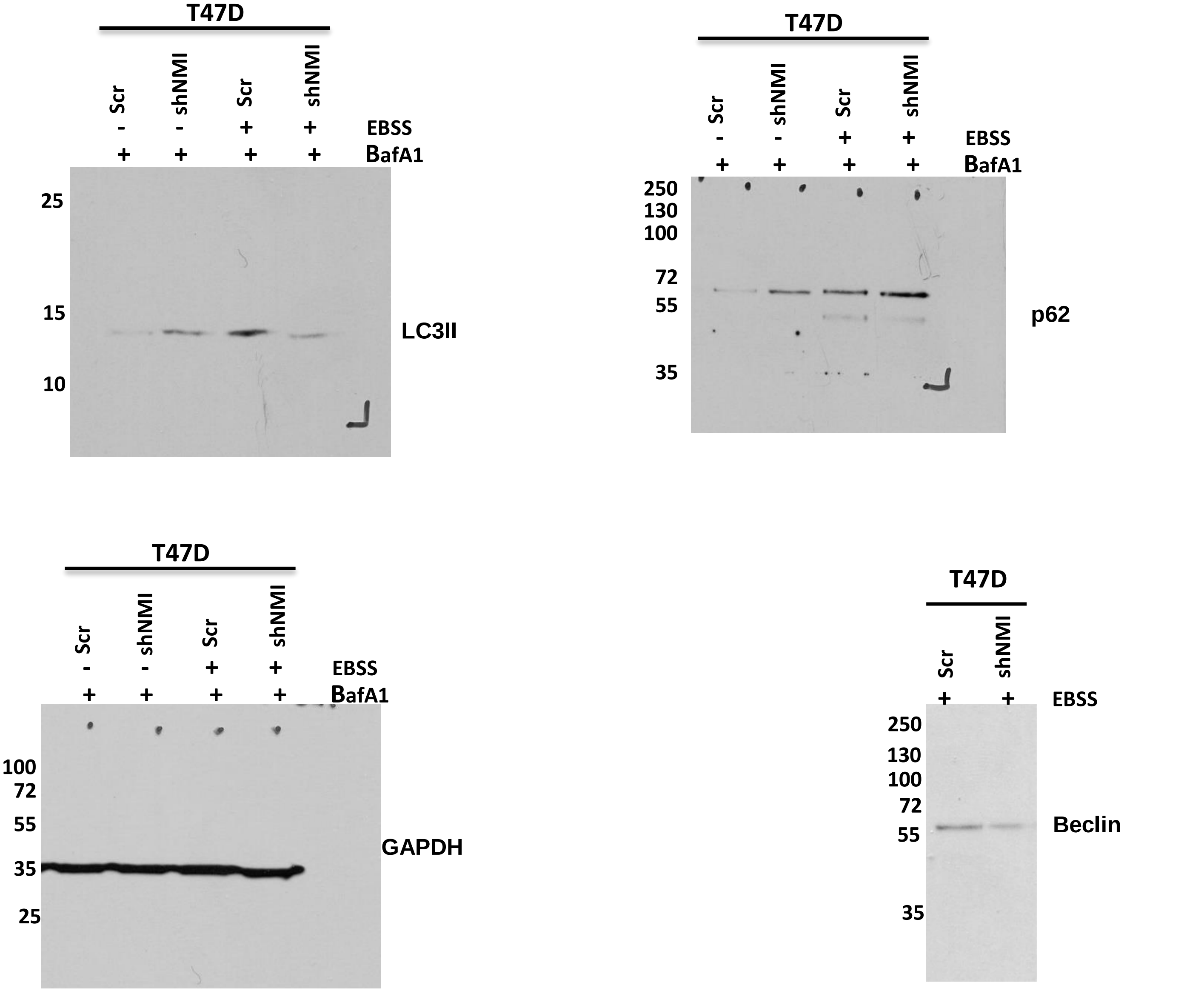

Supplementary Figure 3

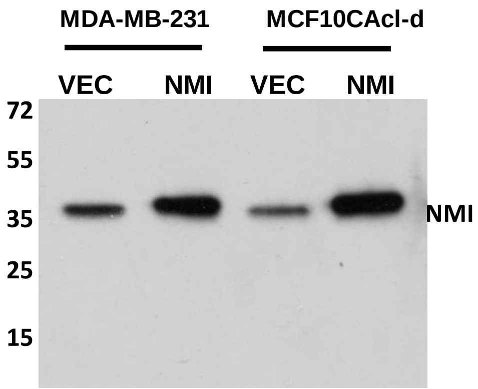

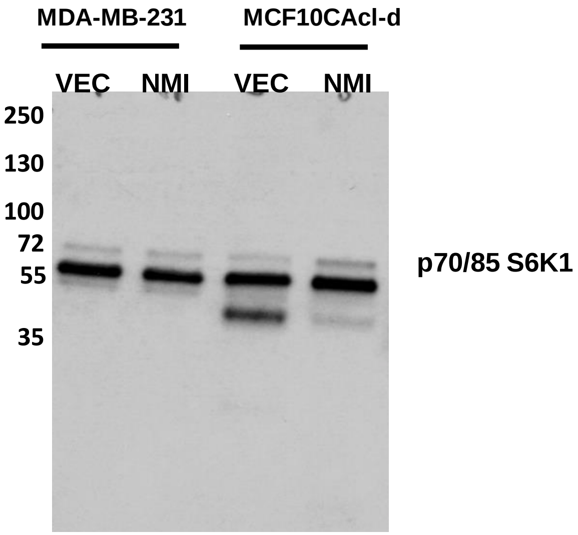

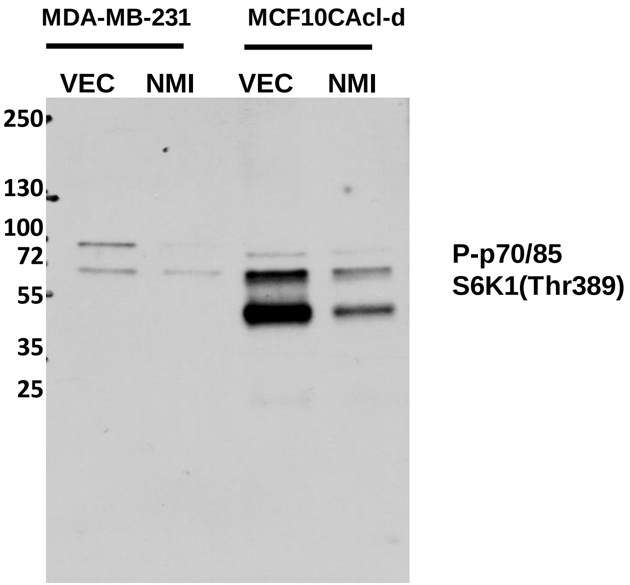

Supplementary Figure 4

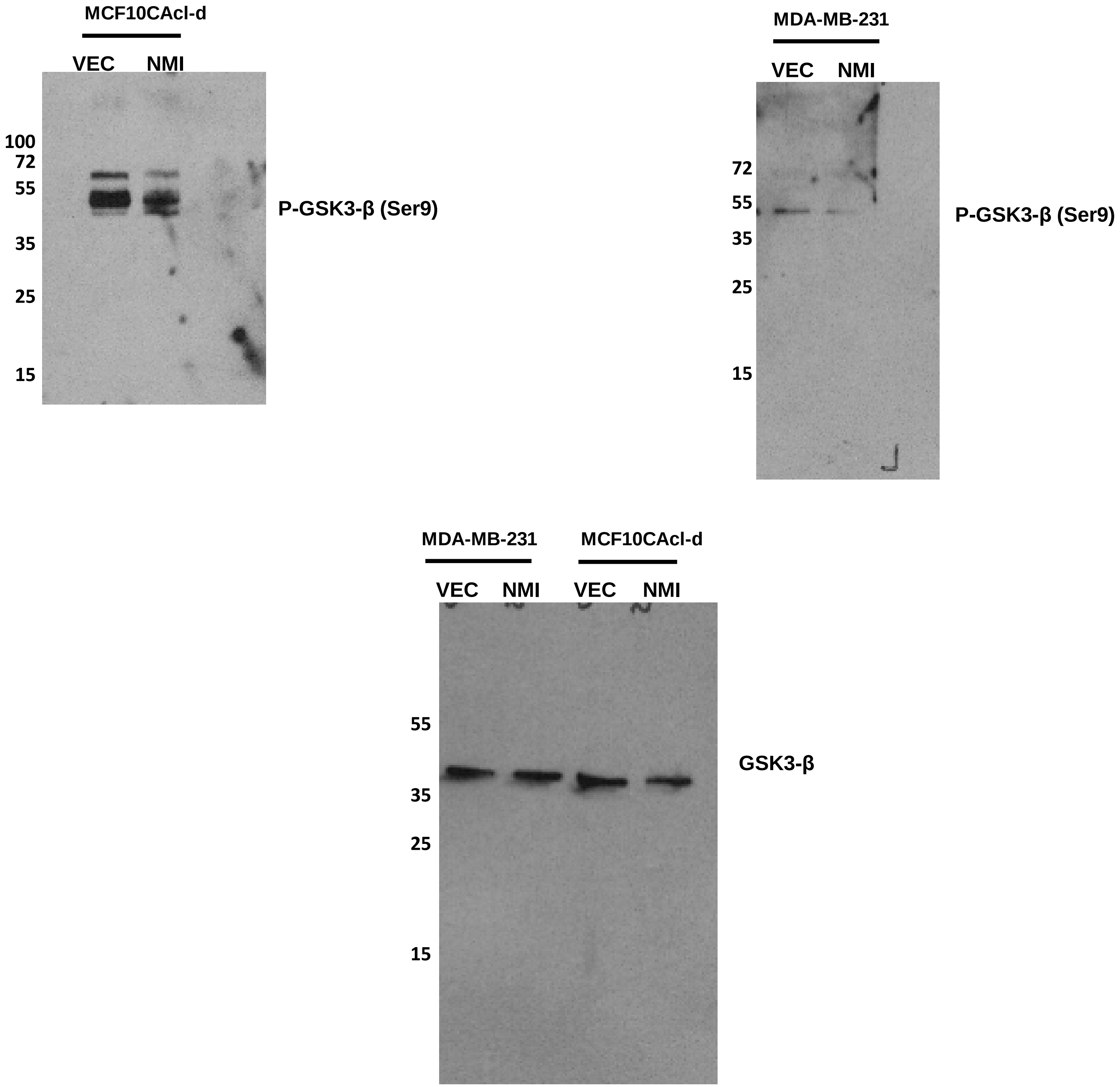

Supplementary Figure 5

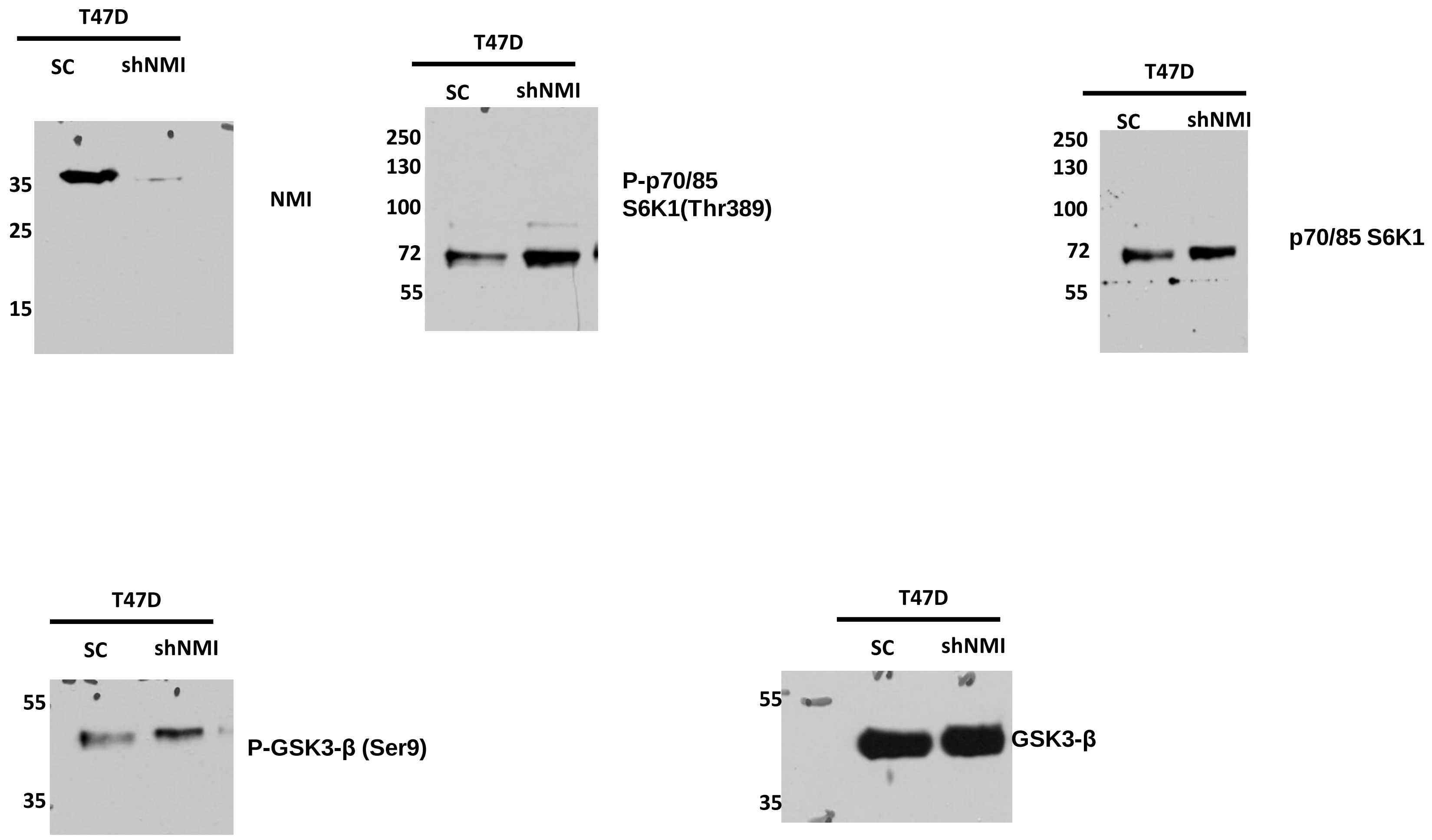

Supplementary Figure 6

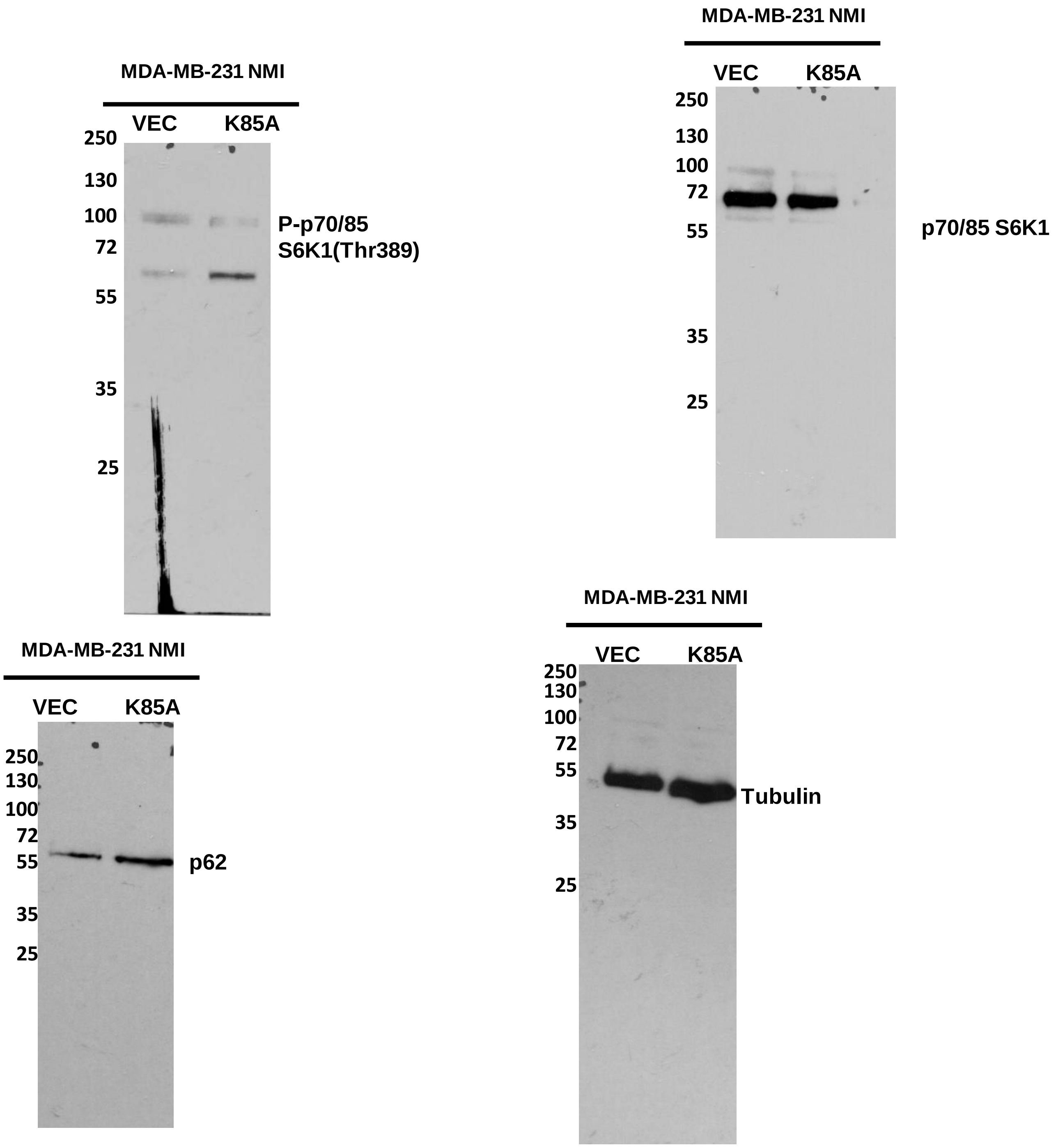

Supplementary Figure 7

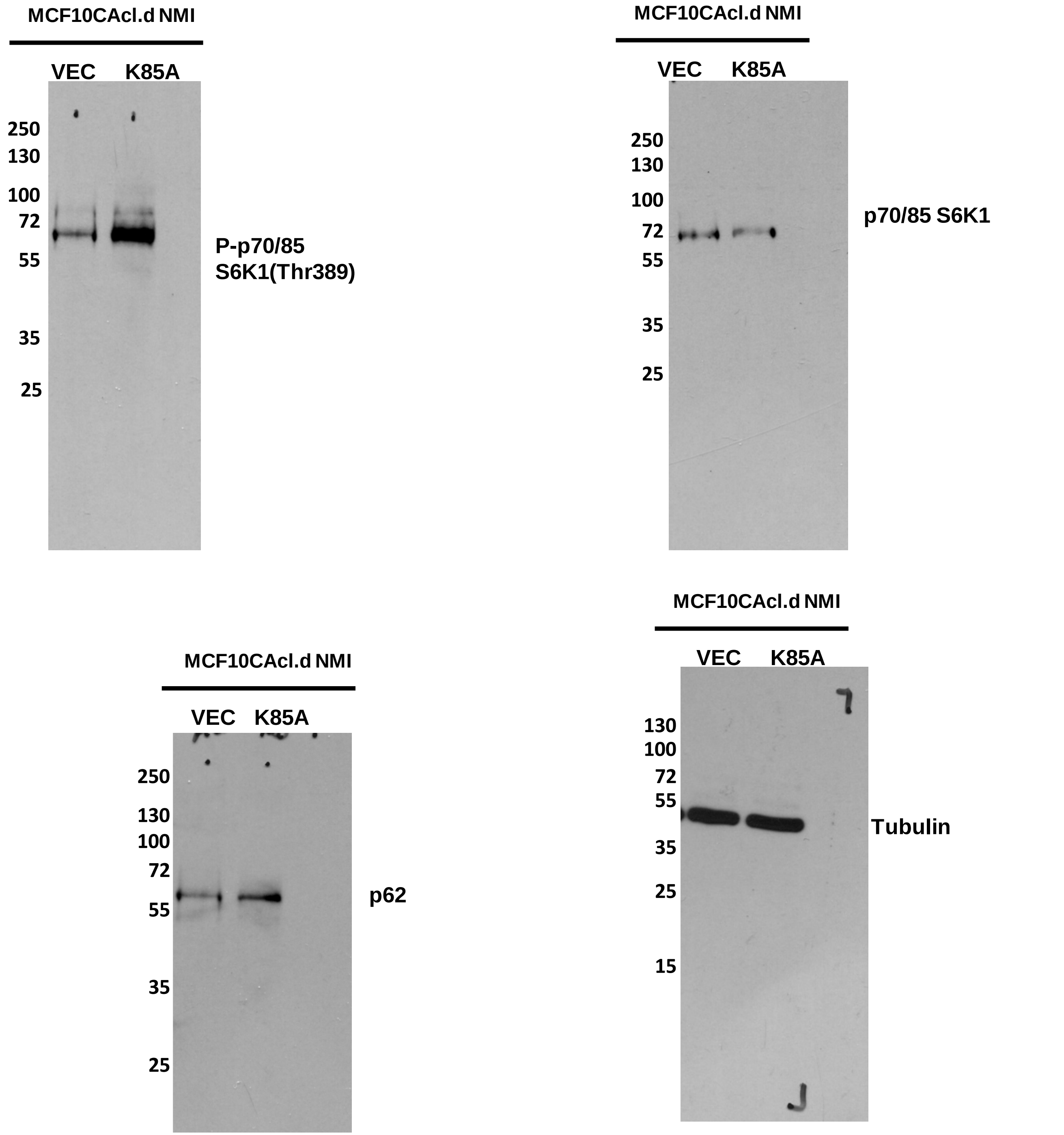

Supplementary Figure 8

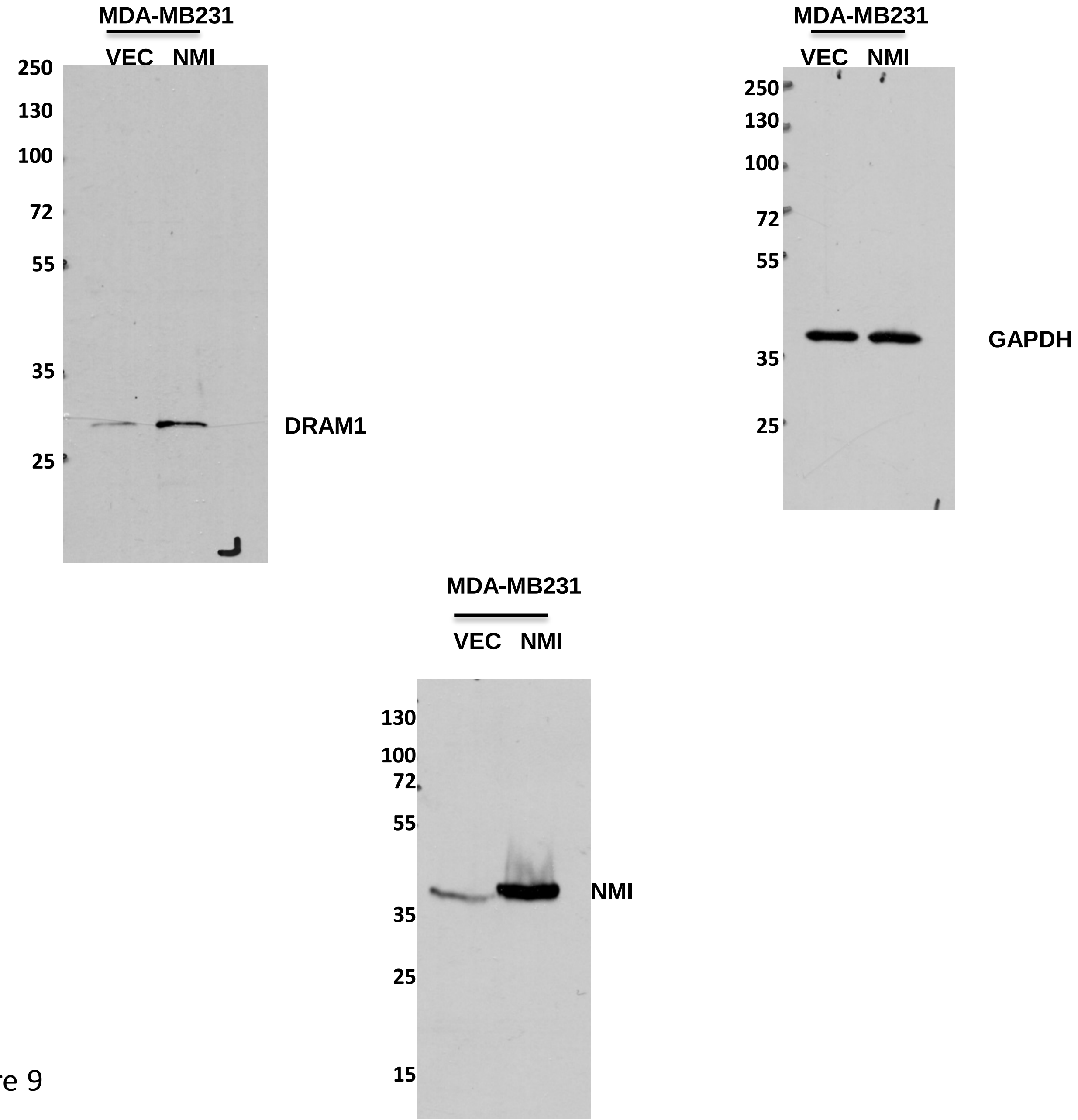

Supplementary Figure 9

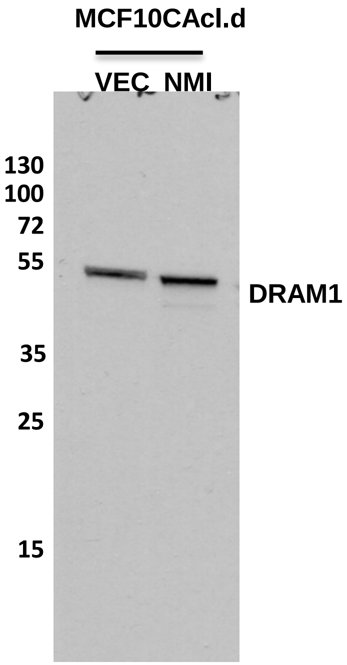

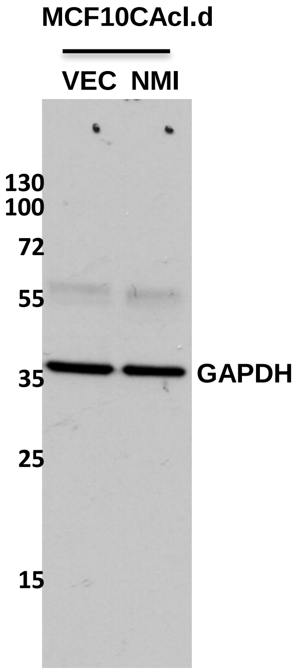

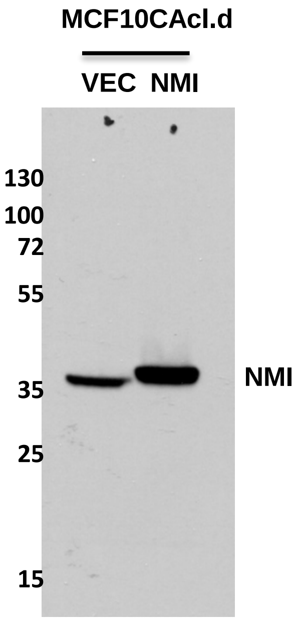

Supplementary Figure 10

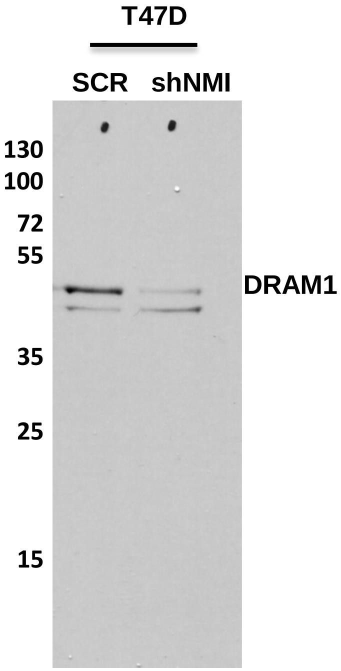

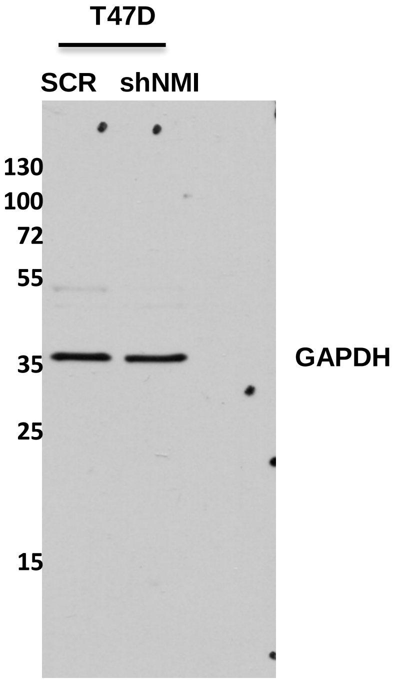

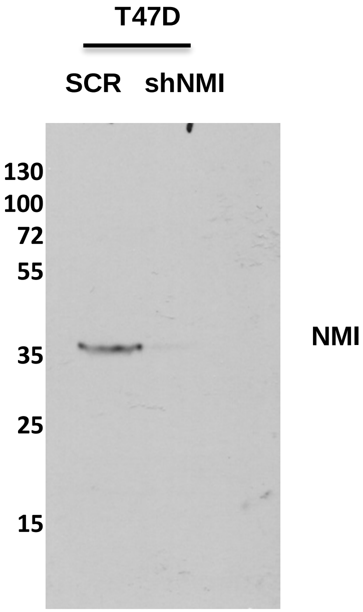

Supplementary Figure 11

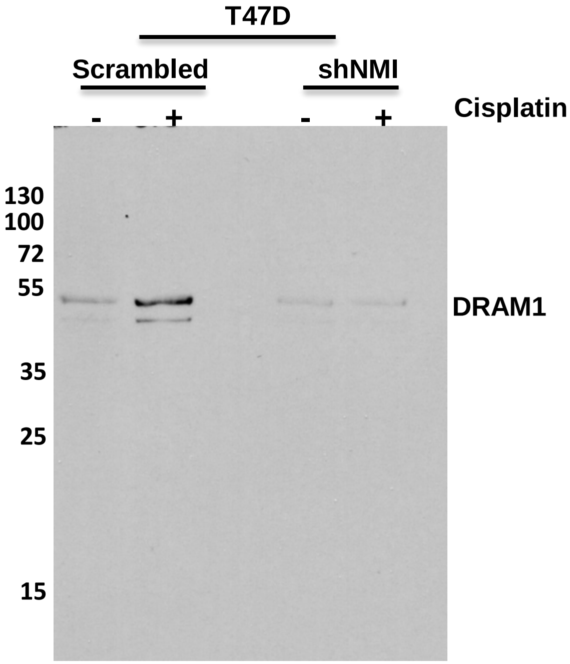

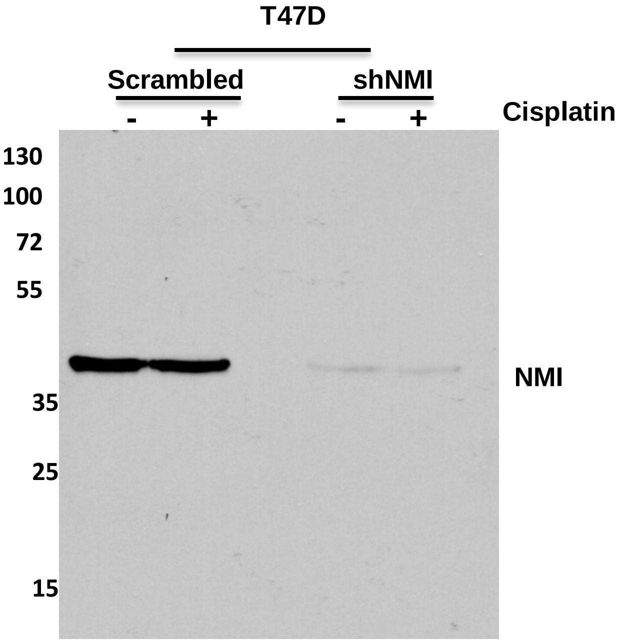

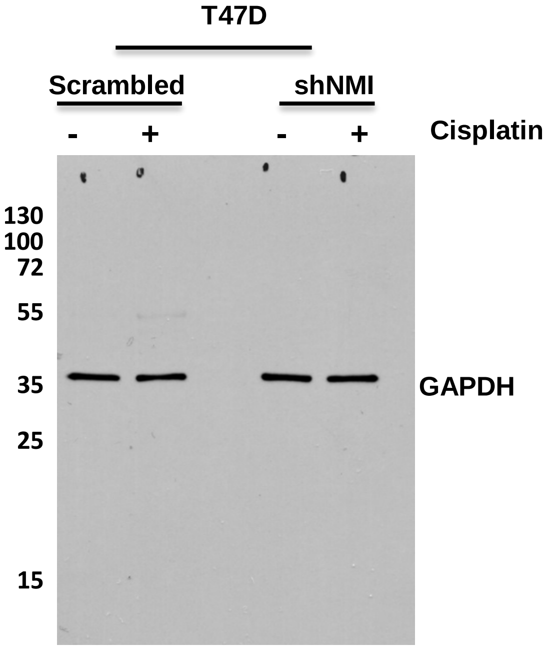

Supplementary Figure 12

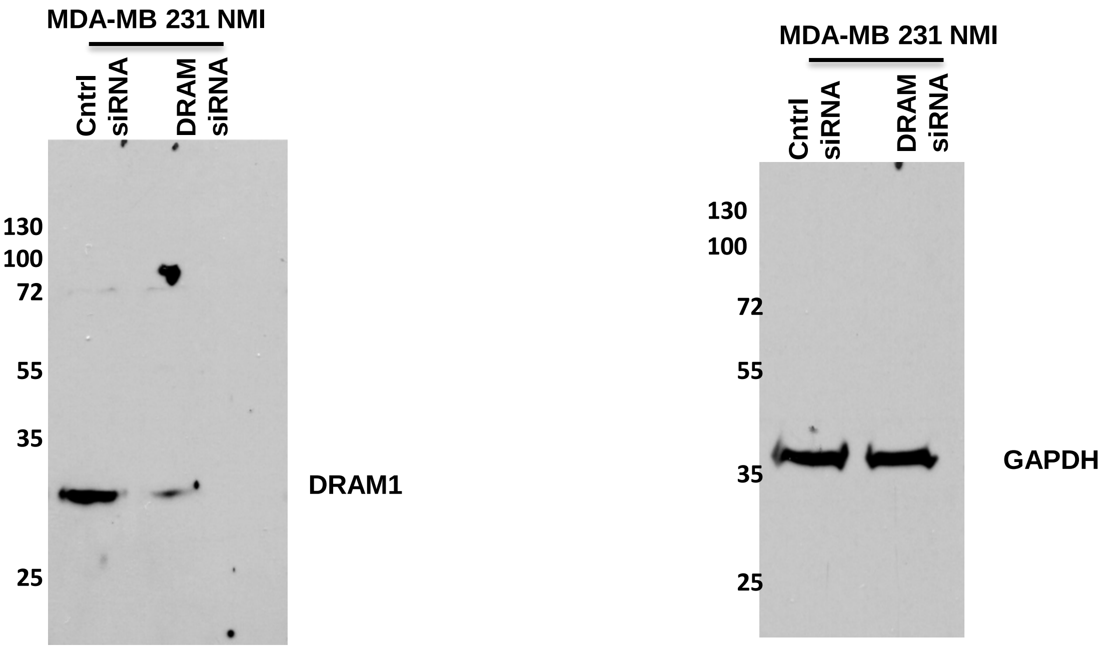

Supplementary Figure 13
